# Behcet’s disease in budd-chiari syndrome

**DOI:** 10.1186/s13023-014-0153-1

**Published:** 2014-09-13

**Authors:** Anne Claire Desbois, Pierre Emmanuel Rautou, Lucie Biard, Nadia Belmatoug, Bertrand Wechsler, Mathieu Resche-Rigon, Virginie Zarrouk, Bruno Fantin, M Pineton de Chambrun, Patrice Cacoub, Dominique Valla, David Saadoun, Aurélie Plessier

**Affiliations:** Department of Internal Medicine and clinical Immunology APHP, Paris France, Centre de référence des maladies autoimmunes et systémiques rares, Université Pierre et Marie Curie, Paris 6, Paris, France; Laboratory I3 “Immunology, Immunopathology, Immunotherapy”, UMR CNRS 7211, INSERM U959, Groupe Hospitalier Pitié-Salpetrière, DHU I2B Inflammation, Immunopathology, Biotherapy, Université Pierre et Marie Curie, Paris 6, Paris, France; Department of Hepatology; Hôpital Beaujon, INSERM U773, Service d’hépatologie, 100 boulevard du Général Leclerc, 92118 Clichy cedex, France; Department of Internal Medicine, Hôpital Beaujon, Clichy, France; Department of Biostatistics and Medical Data Processing; INSERM U717, Hôpital Saint-Louis, Paris, France; Université Pierre et Marie Curie-Paris 6, Paris, F-75013 France; AP-HP, Hôpital Pitié-Salpêtrière, Service de Médecine Interne et d’Immunologie clinique, F-75013 Paris, France

**Keywords:** Budd-chiari syndrome, Hepatic vein thrombosis, Behcet’s disease, Immunousuppressive agents

## Abstract

**Background:**

Behcet’s disease (BD) is a well-known cause of Budd-Chiari syndrome (BCS). Data are lacking on the presentation and outcome of BCS related to BD.

**Methods:**

We investigated the relationship between BD and BCS in 14 patients with both diseases and compared the results to 92 BCS patients without BD.

**Results:**

Male gender (p = 0.003), North African origin (*P* = 0.007) and inferior vena cava obstruction (*P* < 0.0001) were more frequent in patients with BD and BCS than in those with BCS alone and the plasma C-reactive protein level was higher (p = 0.003). Two of the patients with the combined diseases underwent recanalization of the vena cava and the hepatic veins, none received transjugular intrahepatic portosystemic shunts (TIPS), one received a surgical shunt and one underwent liver transplantation. TIPS were less frequent in patients with BD and BCS than in those with BCS alone (*P* = 0.019). Eighty six per cent of patients with BCS and BD received corticosteroids and immunosuppressive therapy. The 5-year transplantation-free survival rate was 63% in patients with BCS alone and 91% in those without BD (*P* = 0.11). In our series and in the literature, a high number of patients [12 (61.5%) and 11 (64.7%) respectively] treated with anticoagulation and corticosteroids and/or immunosuppressants did not require invasive treatment.

**Conclusion:**

This study shows a higher frequency of IVC obstruction in patients with BCS and BD. Medical treatment with anticoagulation and immunosuppressive agents may improve the symptoms of BCS. Therefore early management with immunosuppressive and anticoagulation therapy appears to be the treatment of choice in patients with BCS and BD.

## Background

Budd Chiari Syndrome (BCS) is related to an obstruction of the hepatic venous outflow tract at the hepatic veins or the inferior vena cava (IVC). This condition is associated with a high risk of complications and death due to portal hypertension and liver failure. Most cases of BCS are related to thrombosis resulting from one or several prothrombotic conditions [1]. Myeloproliferative disorders are associated in approximately 50% of cases, and represent the leading cause of primary BCS. Other acquired [e.g. antiphospholipid syndrome (about 10%) or paroxysmal nocturnal hemoglobinuria (about 2%)] and inherited conditions are the cause in a smaller proportion of BCS. Behcet’s disease (BD) is found in approximately 5% of patients with BCS in western countries [1]. BD was reported in 9% of BCS patients in Turkey, making it the third cause of the disease in that country [2], and 13% of patients in Egypt [3].

BD is a chronic and relapsing vasculitis characterized by oral and genital aphtosis, ocular inflammation as well as cutaneous, vascular and nervous system manifestations [4]. Vasculitis is the main pathological finding of BD, and all sized vessels can be involved in the arterial and venous systems [5-7], although venous involvement is more frequent than arterial. In a recent survey, 37% of BD patients had venous thrombosis, and BCS occurred in 2.4% of these cases [8]. BCS is the most severe venous manifestation of BD and is associated with a 9 fold-increase in mortality in these patients [8].

Because BD and BCS are uncommon, data on BCS related to BD are limited to small retrospective series or single case reports. Thus data on the clinical features and the outcome and management of patients with BCS and BD are lacking. The present study retrospectively compared 14 patients with both diseases to 92 patients with BCS without BD.

## Methods

### Patients

The characteristics of 14 consecutive BCS patients with BD diagnosed between 1995 and 2012 were compared to those of a previously reported cohort of 92 BCS patients without BD, diagnosed between 1995 and 2005 [9]. All fourteen patients with BCS and BD met the ICBD criteria for BD [4]. BD patients were followed in both departments [the department of internal medicine at Hôpital Pitié-Salpêtrière (Paris, France) and the liver unit at Hôpital Beaujon (Clichy, France) for BCS management]. The patients with BCS without BD were followed at the Hepatology Department of the Hôpital Beaujon. The diagnosis of BCS was based on confirmation of hepatic venous outflow tract obstruction based on imaging data: venous Doppler ultrasound, CT angiography and/or magnetic resonance angiography (MRA).

Venous outflow obstruction due to right-sided heart failure and sinusoidal obstruction syndrome were not included.

Data on demographic characteristics were collected during the study period (gender, age and geographic origin), clinical presentation of BCS (date of BCS, main symptoms, number of occluded veins), risk factors for thrombosis [proteins C, S and antithrombin III deficiency R506Q mutation of factor V, G20210A mutation of prothrombin and C677T mutation of methylene-tetrahydrofolate-reductase (MTHFR) gene, plasma homocysteine level, anticardiolipin and anti-β2Gp1 antibodies, tests for paroxysmal nocturnal hemoglobinuria and JAK2 V617F mutation] and laboratory findings (liver enzymes, serum creatinine, prothrombin time) and imaging features. Search for signs of BD were recommended as part of the routine work-up. Treatment and overall survival was recorded for each patient. The Rotterdam prognostic score was calculated according to Murad et al. [10].

### Statistical analysis

Patient characteristics and survival were compared between BCS patients with and without BD. Quantitative variables are reported as medians and ranges and were compared by the Wilcoxon rank-sum test. Categorical variables were described with counts and percentages and were compared by the Fisher’s exact test. Overall survival was defined as the time between the date of diagnosis of BCS and death. Living patients were censored at the date of the last recorded follow-up visit. Overall survival was estimated using the Kaplan Meier estimator and compared between groups by the log rank test. The probability of survival was presented as a percent and a 95% confidence interval. All tests were two-sided and *P*-values ≤0.05 were considered to be significant. Analyses were performed using the R statistical software version 2.14.0 (available online at http://www.R-project.org).

### Review of the literature

We systematically reviewed the medical literature via PubMed using the following keywords: “Budd-Chiari Syndrome”, “Behcet’s disease” and “hepatic vein occlusion”. We only analyzed cases reports and series published after 1980 in English or in French.

## Results

### Baseline characteristics

Baseline characteristics of the 106 patients with BCS with and without BD are summarized in Table 1. Fourteen BCS patients also had BD. The median age at diagnosis of BCS was 33 years old (range 22–45) and 79% were men. Male gender (p = 0.003) and North African origin (p = 0.007) were more frequent in patients with BCS and BD than in those without BD (n = 92).

**Table 1:** **Baseline characteristics of BCS patients with and without BD**

	**BCS with BD (n =14)**	**BCS without BD (n =92)**	***p***
Age at BCS diagnosis, median (range)	33 (22–45)	38 (16–77)	0.086
Male gender, *n* (%)	11/14 (79%)	32/92 (35%)	**0.003**
Geographic origin			**0.0002**
Europe	6 (43%)	66 (73%)	
North Africa	8 (57%)	12 (13%)	
Type of outflow obstruction			
IVC occlusion, *n* (%)	10/14 (71%)	16/91 (18%)	**< 0.0001**
HV occlusion alone, *n* (%)	4/11 (36%)	75/91 (82%)	0.002
Combined HV and IVC occlusion, *n* (%)	6/11 (55%)	15/91 (16%)	**0.009**
Number of hepatic veins thrombosed, median ± SD	2	3	**0.017**
1 HV thrombosed, *n* (%)	3/10 (30%)	5/91 (5%)	
2 HV thrombosed, *n* (%)	1/10 (10%)	13/91 (14%)	
3 HV thrombosed, *n* (%)	5/10 (50%)	72/91 (79%)	
Clinical features at baseline^a^, *n* (%)			
Ascites	6/13 (46%)	72/92 (78%)	0.036
Hepatomegaly	6/13 (50%)	55/92 (60%)	0.38
Splenomegaly	2/12 (17%)	51/92 (55%)	**0.014**
Abdominal pain	6/13 (46%)	62/92 (67%)	0.21
Hepatic encephalopathy	0/13	7/90 (8%)	0.59
Gastrointestinal bleeding	0/13	10/92 (11%)	0.36
Laboratory at baseline^a^			
ALT < 5 × ULN, *n* (%)	10/11 (91%)	60/92 (65%)	0.10
AST < 5 × ULN, *n* (%)	10/11 (91%)	63/92 (68%)	0.17
Bilirubinemia (μmol/l), med(min-max)	13 (6–38)	31 (7 – 207)	**< 0.0001**
Albuminemia (g/l), med(min-max)	37(26–49)	35(19–52)	0.94
CRP level (mg/L), med(min-max)	85(7–238)	16(3–344)	**0.003**
Creatinemia (μmol/L), med(min-max)	80(44–120)	73(36–428)	0.34
Prothrombin Time < 70%, *n* (%)	5/11 (45.5%)	62/92 (67.4)	0.19
Thrombopenia (< 150.10 [9]/L), *n* (%)	2/12 (16.7%)	18/92 (19.6%)	1
Rotterdam BCS index	1 (0.0;1.2)	1.2 (0.0;3.9)	0.0007

*Abbreviations*: *ALT* Alanine aminotransferase, *AST* Aspartate aminotransferase, *BD* Behcet’s disease, *BCS* Budd-Chiari Syndrome, *CRP* C reactive protein, *HV* Hepatic vein, *IVC* Inferior vena cava, *Med* Median, *ULN* Upper limit of normal.

^a^In BD patients, laboratory values at baseline were available in 11 patients for AST, ALT, creatinemia and CRP level, in 10 patients for albuminemia and in 12 patients for biliburinemia.

In BCS patients without BD, laboratory values were available in 90 patients for bilirubinemia, in 73 patients for albuminemia, in 87 patients for creatinemia and 41 patients for CRP level.

Bold indicates significant differences.

Four (28.6%) patients had already been diagnosed with BD when BCS was diagnosed [median time 11 months (range 3–97 months)]. The diagnosis of BD was concomitant with the diagnosis of BCS in 8 (57%) patients. In the remaining 2 patients, BD was diagnosed 14 and 30 months after BCS, respectively. Major organ or tissue involvement of BD included oral (100%) and genital mucosa (64%), eyes (29%), joints (29%), nervous system (21%), arterial bed (29%) and heart (50%).

Ascites and splenomegaly were less common in BCS patients with BD than in those without (Table 1). Median C-reactive protein (CRP) levels at the diagnosis of BCS were higher in BD patients than in those without. Laboratory results were similar in both groups, except for a lower serum bilirubin level in BD patients. IVC obstruction (suprahepatic and infrahepatic IVC occlusions) at presentation was almost 4 times more common in BD patients with BCS than in those without BD. Most (n =11, 79%) patients with BCS and BD had other types of venous thrombosis as described in Table 2. Most of these thromboses were diagnosed at the same time as BCS in BD patients. Five patients (36%) with BCS and BD had an associated prothrombotic condition (Table 2). Three out of 14 BD patients [21.4%, 95% CI (7.1; 48.5)] were HLA B51 positive.

**Table 2:** **Disease characteristics of patients with BD and BCS**

	**Patients with BD and BCS (n = 14)**
Additional etiologic factors, *n* (%)	5 (38.5%)
Antiphospholipid antibodies	2
Hyperhomocyteinemia	2
Factor II heterozygous gene mutation	1
Associated venous thrombosis, *n* (%)	
Pulmonary embolism	7 (50%)
Intracardiac [right atrium/right ventricle]	5 (36%) [29%/7%]
Superior vena cava	3 (21%)
Lower limbs	4 (29%)
Cerebral	1 (7%)

*Abbreviations*: *BCS* Budd-Chiari Syndrome, *BD* Behcet’s disease.

### Treatments of BCS in BD patients

**Figure 1:**
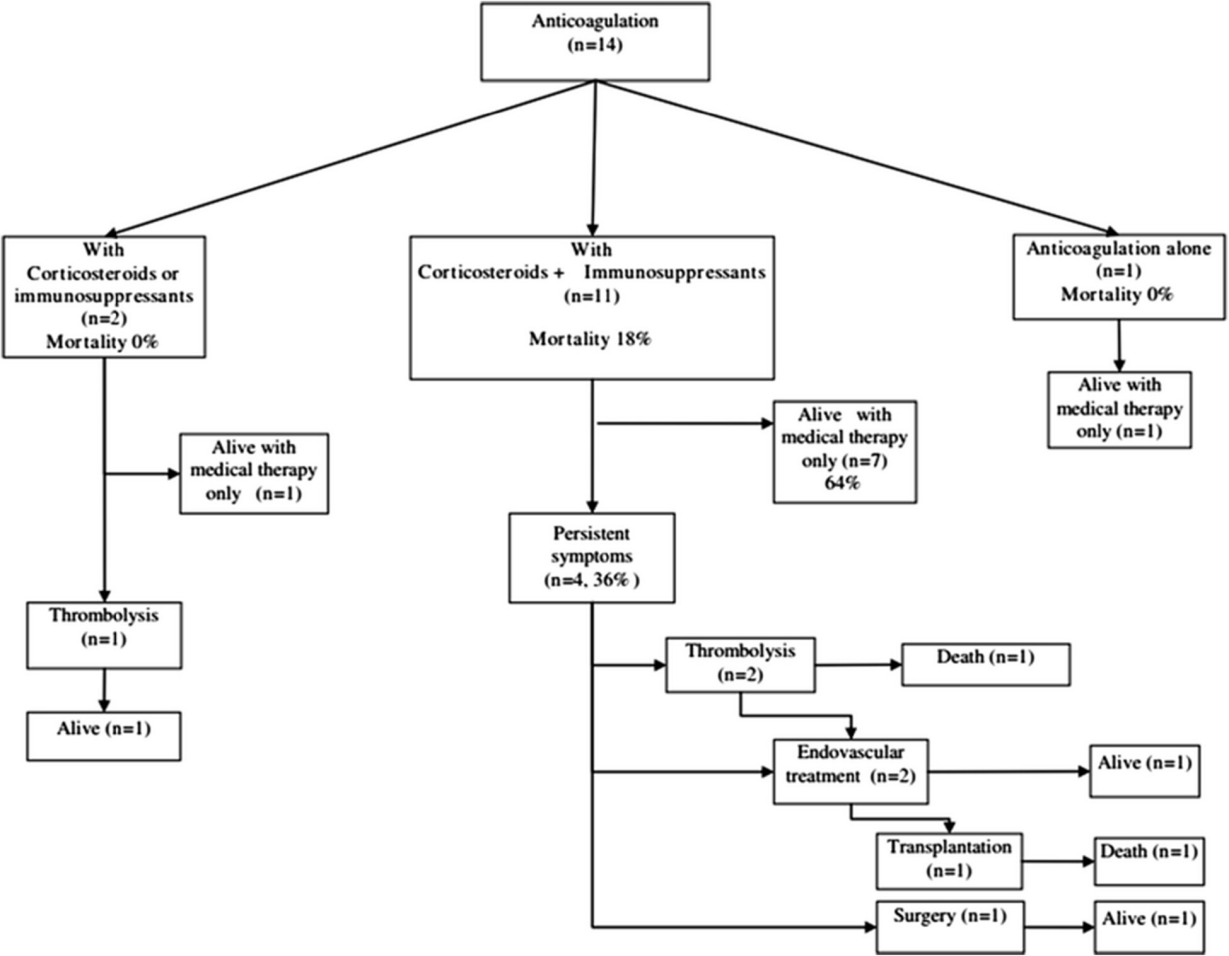
**Outcome of the 14 BD patients according to treatments (specific treatments of BD and treatments of BCS).**

The main features of BCS related treatment are indicated in Table 3 and Figure 1. Treatment included anticoagulation with heparin followed by a coumarine derivative in all cases. None of the BCS patients with BD received transjugular intrahepatic portosystemic shunts (TIPS) and one underwent liver transplantation. Patients with BCS and BD received TIPS less frequently and vena cava thrombolysis more often than those without BD (Table 3). The reason that TIPS were not used included a good response to medical therapy in 11 patients with BCS, or a contraindication due to associated IVC obstruction in 3.

**Table 3:** **Main treatments of BCS patients with and without BD**

	**BCS with BD (n =14)**	**BCS without BD (n =92)**	***p***
Anticoagulation, *n* (%)	14 (100%)	92 (100%)	1.00
Endovascular treatment, *n* (%)	2 (14%)	17 (18%)	1.00
Thrombolysis, *n* (%)	3 (21%)	1 (1%)	**0.007**
TIPS, *n* (%)	0 (0%)	28 (30%)	**0.019**
Surgical decompression^a^, *n* (%)	1 (8%)	7 (8%)	1.00
OLT, *n* (%)	1 (7%)	15 (16.3%)	0.69

*Abbreviations*: *BCS* Budd-Chiari Syndrome, *BD* Behcet’s disease, *TIPS* Transjugular intrahepatic portosystemic shunt, *OLT* Orthotopic liver transplantation.

^a^Data on surgical decompression was available for 13 patients in BCS patients with BD.

Bold indicates significant differences.

Complications of anticoagulation included epistaxis and hematuria in 1 patient.

### Treatment of BD

Specific treatment for BD is presented in Figure 1. Twelve (86%) patients with BCS and BD received corticosteroids (0.5-1 mg/kg/day of oral prednisone in 12 patients including pulses of 1 g methylprednisolone for 3 days in 2 cases). Twelve (86%) patients received immunosuppressive therapy including azathioprine (2–3 mg/kg/day, n = 9), cyclophosphamide (pulses of 750 mg/m [2] /4 weeks during 6–12 months, n = 4), oral cyclosporine (3 mg/kg/day, n = 1). One patient received anti-tumor necrosis factor-α (anti-TNFα) inhibitor (infliximab 5 mg/kg intravenously at week 0, 2, 6 and every 6 to 8 weeks, n = 1). Three patients received 2 immunosuppressive and/or biologic agents [cyclophosphamide followed by azathioprine (n = 1) or cyclosporine (n = 1) and infliximab followed by azathioprine (n = 1)].

Two (15.4%) of the 13 patients who received anticoagulation and corticosteroids and/or immunosuppressants died; immunosuppressive therapy was started more than 2 years after the diagnosis of BCS in 1 of these. Eight (61.5%) of these 13 patients did not require endovascular treatment or surgery. The 5 other patients required additive invasive treatments [thrombolysis (n = 3), additional stent (n = 1) and surgical decompression by mesoatrial shunting (n = 1)]. This last patient was still alive after surgery at the end of follow up but had refractory ascites.

The patient who received only anticoagulation had a favourable outcome.

The main complications of immunosuppressive agents included adenitis with azathioprine (n = 1), zoster infection with anti-TNF alpha therapy (n = 1) and pancytopenia under azathioprine (n = 1).

### Survival

**Figure 2:**
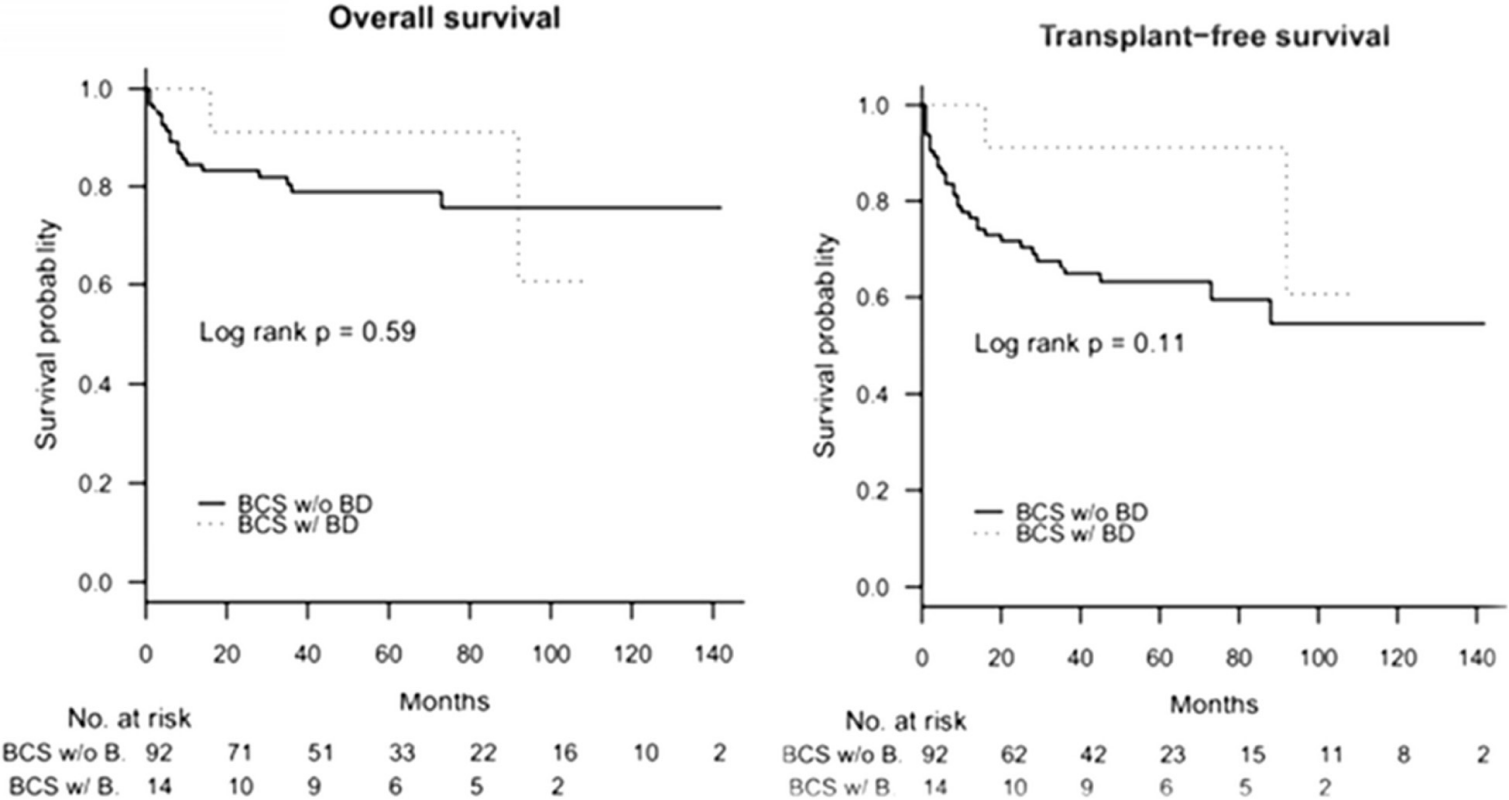
**Overall survival in BCS patients with (n = 14) and without (n = 92) BD (a); Transplantation free survival in BCS patients with (n = 14) and without (n = 92) BD (b).**

Median follow-up for the study group (n = 106) was 54 months (range 1–142 months). Mortality in BCS patients with BD was 14.3% after a median follow up of 53 months. One and 5-year overall survival rates were 84% (CI 95% 77–92) and 79% (CI 95% 71–88) respectively in BCS patients without BD; and 100% and 91% (CI 95% 75–100) respectively in BCS patients with BD (no significant difference) (Figures 2a and b). Transplantation free survival rates in BCS patients with and without BD, were 100% and 77% (CI 95% 68–86) at 1 year, and 91% (CI 95% 75–100) and 63% (CI 95% 53–75) at 5 years, respectively.

Death was related to liver disease in the 2 patients with BD who died.

### Literature review

Out of the 95 BD patients with BCS reported in literature, the 61 from publications in English and French were included in the study [11-32]. Data were obtained from 19 case-reports including fewer than 5 patients) and 3 series (including more than 5 consecutive patients). Ninety percent of the patients were men. Median age was 26 years old (range 12–58). All patients were symptomatic. Ninety eight percent of the patients had ascites, 74% hepatomegaly, 26% splenomegaly, 23% abdominal pain, 16% jaundice and 13% hepatic encephalopathy. Most patients had elevated liver enzymes. The mean serum bilirubin level was 79 μmol/L, and the mean prothrombin time ratio was 49%. Ninety one percent of the patients had associated IVC thrombosis. Lower limb thrombosis, intracardiac thrombosis, pulmonary embolism and renal vein thrombosis were associated with BCS in 28, 22, 13 and 9% of the patients, respectively.

Treatment and outcome were available in 32/61 patients with BCS and BD reported in literature. Data are summarized in Table 4. After a mean follow-up of 30 months, 19 (59%) patients improved, 11 (34%) died, 1 relapsed and 1 had persistent ascites. Two (12%) of the 17 patients treated with anticoagulation, corticosteroids and/or immunosuppressive agents, died (despite surgical decompression in one), 11 (65%) improved without receiving endovascular/surgical treatment, 2 improved after surgical or endovascular treatment, 1 had persistent ascites and 1 relapsed 5 years after surgery. Four (67%) of the 6 patients treated with corticosteroids with or without immunosuppressants and without anticoagulation died and 2 had a favourable outcome. Three of the 5 patients (60%) treated with anticoagulation only died (despite surgical decompression in one case) and 2 were still alive in good physical condition after thrombectomy in one [14]. Two of the 4 patients who did not receive anticoagulation, corticosteroids or immunosuppressive agents died (including one after thrombectomy) and the remaining 2 cases improved [after intravenous thrombolysis (n = 1) and with aspirin and colchicine (n = 1)] [11].

**Table 4:** **Treatments and outcome of the 61 BD patients with BCS reported in literature**

**Date of publication/authors**	**Number of patients**	**Anti-coagulation**	**Cortico-steroids**	**Immuno-suppressive therapy**	**Surgery**	**Endovascular treatment**	**Outcome**	**Follow up (months)**
1980/ [11]	1	0	0	0	0	0	death	4
1996/ [20]	1	0	0	0	1(thrombectomy)	0	death	During surgery
2000/ [23]	1	0	0	0	0	0	favourable	180
1983/ [13]	1	T	0	0	0	0	favourable	72
No treatment	4	0 (T, n =1)	0	0	1 thrombectomy	0	Death n = 2 (50%)	
1985/ [14]	1	0	1	1	0	0	favourable	24
2002/ [24]	1	0	1	1	0	0	death	3
2007/ [28]	1	0	1	0	0	thrombolysis (failure)	death	ND
2007/ [29]	3	0	3	3	0	0	death (n = 2) favourable (n = 1)	7;7;6
No ATCG	6	0	6	5	0	0	Death n = 4 (67%)	
1983/ [12]	1	1	0	0	1(shunt)	0	death	1
1986/ [15]	1	1	0	0	0	1 (stent)	favourable	36
1991/ [18]	1	1	0	0	0	0	death	1.25
1990/ [17]	1	1	0	0	0	0	favourable	24
2008/ [30]	1	1	0	0	0	0	death	ND
ATCG alone	5	5	0	0	1	1	Death n = 3 (60%)	
1990/ [17]	3	3 (T, n = 1)	2	1	2 (shunt)	0	death (n = 1)	30;4;4
2002/ [25]	1	1	1	0	1	0	relapse (n = 1)	60
2004/ [26]	1	1	1	1	0	dilatation and stent	favourable	0.25
2007/ [27]	4	4	4	4	0	0	death (n = 1)	1;96;48;36
2008/ [30]	6	6	6	2	0	0	favourable (n = 6)	43
2011/ [31]	1	1	1	1	0	0	favourable	1
2011/ [32]	1	1	1	1	0	0	alive	1.5
ATCG and IS	17	17	16	10	3	1	Death n = 2 (12%)	
TOTAL	32	ATC 22 (69%)	22 (69%)	15 (47%)	5 (16%)	3 (9%)	12 (38%)	29.7 months
		Thrombolysis 2 (6%)						

*Abbreviations*: *ATC* Anticoagulation, *IS* Immunosuppressants, *ND* No determined, *PT* Prothrombin time, *T* Thrombolysis.

## Discussion

This study reports the largest series of consecutive patients with BCS and underlying BD. Most previous studies of this rare combination of disorders have consisted of case reports and therefore little is still known about the specific clinical features, treatment outcome or prognosis. In the current study, we compared the course of the disease in 14 patients with BCS and BD to a cohort of patients with BCS in whom BD was excluded as the underlying cause. Previous surveys indicate that BD may be responsible for up to 13% of the cases of BCS [1-3].

The results of this study confirm and expand those from previous smaller studies [3,17] on baseline characteristics of patients at diagnosis of BCS. The main clinical features at diagnosis of BCS that differed between patients with and without BD were a younger age, a significant predominance of men, a North African origin and a higher frequency of associated thrombosis in other territories, as expected for BD patients in general. These findings are similar to the data from Bismuth et al. who reported male predominance (male/female ratio, 19:1) in BCS patients with BD compared to those without (i.e. mainly patients with myeloproliferative disorders) [17].

Our study reported a 4-fold higher prevalence of IVC thrombosis in patients with than in those without BD. This emphasizes the significant association between BCS and IVC occlusion in patients with BD. Thus, the presence of IVC thrombosis in patients with BCS should suggest BD, which should then be investigated. Indeed, the diagnosis of BD had not yet been made in 2/3 of BCS patients with BD when BCS occurred. In our study, most of patients with BCS and BD had thrombosis in other territories (i.e. pulmonary artery, intracardiac, superior vena cava) which might be a useful indication to suggest and search for BD.

Despite a higher frequency of associated IVC thrombosis, the short-term prognosis of BCS patients with BD did not differ from that in BCS patients without BD. The presence of associated IVC thrombosis in BD patients and the favourable outcome under immunosuppressants explain in part the low frequency of TIPS. Bayraktar et al*.* [22] observed a better outcome in BD patients with BCS without IVC thrombosis (100% survival in 2 patients) while the mortality was 66% in patients with BCS and IVC thrombosis (n = 8) . In our cohort, none of the 4 patients with BCS and BD without IVC obstruction died. However, in recent surveys on BCS from all causes, IVC obstruction was not found to be associated with death or an intervention in multivariate analysis [33-35].

Early diagnosis of BD is necessary in BCS patients to begin early and specific treatment for BD. Indeed in the present study and in the literature, pharmacological anticoagulation and immunosuppression alone were associated with favourable outcomes in patients with BCS and BD. However, it is important to remember that results in the literature are retrospective and include mainly case-reports, which may cause a significant reporting bias. Moreover, differences with our cohort might also be caused by less uniform management in case-reports. In our survey, the mortality rate in patients treated with anticoagulation and corticosteroids and/or immunosuppressants was 18%. In the literature, the mortality rate in patients with similar treatment was 12% but was 60% in patients treated with anticoagulation only. In our cohort, 8 (62%) patients had a favourable outcome with medical therapy alone (i.e. anticoagulation and immunosuppressive agents and/or corticosteroids) and without endovascular treatment or surgery. In contrast in a large series of BCS from all causes, only 20-49% of patients treated with anticoagulation alone without invasive treatment had a favourable outcome [36,37].

The pathogenesis of thrombosis in BD is not fully understood. Thus far no consistent primary coagulation or fibrinolytic system abnormalities have been identified in BD [38,39]. Because venous inflammation is probably the cause of deep vein thrombosis in patients with BD [40], an immunosuppressive approach to management seems reasonable, although no large randomized controlled trials have directly addressed this issue. In a previous study on venous thrombosis in BD, we showed that immunosuppressive agents significantly improved the prognosis by decreasing the relapse of thrombosis by four fold [8]. Another retrospective survey in 37 BD patients with venous thrombosis compared immunosuppressive, anticoagulation treatment and a combination of immunosuppressants and anticoagulants [41]. Thrombosis recurred in three of the four patients in the anticoagulant treatment group (75%) compared to 2/16 cases (12.5%) in the immunosuppressant group and to 1/17 cases (5.9%) in the combination group.

The limitations of the present study due to the rarity of both conditions must be kept in mind. This was a retrospective analysis and we could not collect complete longitudinal data in patients who were only seen intermittently. Moreover, the studies in the literature are mainly case-reports with a potential selection bias. Nevertheless, this study indicates that (a) most BCS patients with BD present with IVC involvement and other venous thrombosis and (b) TIPS are rarely used (c) despite this, the overall outcome in patients with BD is not different from that in patients with BCS without BD and (d) the association of immune suppression to anticoagulation in medical therapy has a specific impact on BCS in BD patients. Therefore, early combination immunosuppressants and anticoagulation therapy (with caval recanalisation procedures when appropriate), appears to be the treatment of choice in patients with BCS and BD.

## Abbreviations

BCS: Budd-chiari syndrome
IVC: Inferior vena cava
BD: Behcet’s disease
MRA: Magnetic resonance angiography
MTHR: Methylene-tetrahydrofolate-reductase
TIPS: Transjugular intrahepatic portosystemic shunt
CRP: C-reactive protein
ALT: Alanine aminotransferase
AST: Aspartate aminotransferase
